# Rainforest trees respond to drought by modifying their hydraulic architecture

**DOI:** 10.1002/ece3.4601

**Published:** 2018-12-11

**Authors:** David Y. P. Tng, Deborah M. G. Apgaua, Yoko F. Ishida, Maurizio Mencuccini, Jon Lloyd, William F. Laurance, Susan G. W. Laurance

**Affiliations:** ^1^ Centre for Tropical, Environmental and Sustainability Sciences, College of Science and Engineering James Cook University Smithfield Queensland Australia; ^2^ Instituto de Biologia Universidade Federal da Bahia Salvador Bahia Brazil; ^3^ ICREA Pg. Lluís Companys Barcelona Spain; ^4^ CREAF Universidad Autonoma de Barcelona Barcelona Spain; ^5^ Department of Life Sciences Imperial College London Ascot UK; ^6^ Faculdade de Filosofia, Ciencias e Letras de Ribeirao Preto Universidade de Sao Paulo Ribeirao Preto Brazil

**Keywords:** drought, functional anatomy, plant hydraulics, throughfall exclusion, trait plasticity, tropical rainforest

## Abstract

Increased drought is forecasted for tropical regions, with severe implications for the health and function of forest ecosystems. How mature forest trees will respond to water deficit is poorly known. We investigated wood anatomy and leaf traits in lowland tropical forest trees after 24 months of experimental rainfall exclusion. Sampling sun‐exposed young canopy branches from target species, we found species‐specific systematic variation in hydraulic‐related wood anatomy and leaf traits in response to drought stress. Relative to controls, drought‐affected individuals of different tree species variously exhibited trait measures consistent with increasing hydraulic safety. These included narrower or less vessels, reduced vessel groupings, lower theoretical water conductivities, less water storage tissue and more abundant fiber in their wood, and more occluded vessels. Drought‐affected individuals also had thinner leaves, and more negative pre‐dawn or mid‐day leaf water potentials. Future studies examining both wood and leaf hydraulic traits should improve the representation of plant hydraulics within terrestrial ecosystem and biosphere models, and help fine‐tune predictions of how future climate changes will affect tropical forests globally.

## 1 INTRODUCTION

Climate change presents a prominent threat to natural environments (Aragão et al., 2008; Laurance et al., 2009), and one worrisome projection is an increase in periods of extended droughts (Chadwick, Good, Martin, & Rowell, 2016). This is of particular concern for biodiverse, and carbon‐rich tropical rainforests (Allen et al., 2010; Meir & Woodward, 2010), where plants are adapted to relatively high water availability (Sperry, Hacke, Oren, & Comstock, 2002).

The occurrence of longer‐than‐usual drought periods or droughts over an extended number of years can result in widespread seedling mortality (Edwards & Krockenberger, 2006), reduced plant growth (Brando et al., 2008; Corlett, 2016; Laurance et al., 2009), and even large‐scale tree dieback (da Costa et al., 2010; Engelbrecht et al., 2007). Recurring droughts may occur at frequencies that do not give forests time to recover, leading to perpetually damaged ecosystems that can negatively affect the global carbon budget (Schwalm et al., 2017).

Commonly, drought impacts on plants are studied under controlled glasshouse facilities, but this approach falls short in accounting for the complexity of tropical rainforest systems and the responses of mature trees (Meir et al., 2015; Niinemets, 2010). Thus far, ecosystem‐scale rainfall exclusion studies are the only means to assess drought responses of mature trees in situ, as these setups create soil moisture deficit by channeling away a fraction of incoming rainfall (Meir et al., 2015). However, there have only been four such experimental drought setups in tropical forests, including southern Asia (Inoue et al., 2017; Schuldt et al., 2011), South America (da Costa et al., 2010; Nepstad et al., 2002, 2007 ; Nepstad, Tohver, Ray, Moutinho, & Cardinot, 2007), and more recently in Australia (Laurance, 2015).

So far, these experiments have reported changes in growth and mortality at the stand level (da Costa et al., 2010, 2018 ; Fisher et al., 2007; Nepstad et al., 2007), physiological performance (Domingues et al., 2018; Inoue et al., 2017; Rowland, Lobo‐do‐Vale, et al., 2015), or biological traits (Binks et al., 2016b, 2016a; Metcalfe et al., 2010). However, many of these studies have been focused either on an individual species, or worked on the level of genera, and moreover, the plant organ‐level basis of species acclimation, and whether species‐level differences in these responses occur in mature trees, is not nearly as well studied.

In this context, functional traits, or morphological, anatomical or physiological features, may define a set of useful parameters for examining plant strategies to drought and to model the organ‐level changes in plants as they are exposed to environmental stresses (Díaz & Cabido, 2001; Suding et al., 2008; Powell et al., 2017). High wood density, for instance, is often associated with plant drought tolerance (Hacke, Sperry, Pockman, Davis, & McCulloh, 2001), but alone, this trait is too imprecise for describing hydraulic architecture across species (Zanne et al., 2010). Instead, a rapidly growing branch of functional ecology, functional anatomy, emphasizes the utility of wood anatomical characters rather than simply morphological‐based traits (Carlquist, 2001; Martínez‐Cabrera, Jones, Espino, & Schenk, 2009; Zanne et al., 2010). Expressly, the vessel network in tree sapwood and associated tissues that facilitate water transport or storage thus represents the hydraulic architecture of trees (Tyree & Zimmermann, 2002) and these features can therefore provide a framework for understanding species‐specific water‐use strategies, in terms of unique trait combinations within species or trait trade‐offs across species (Apgaua et al., 2015, 2017 ; Worbes, Blanchart, & Fichtler, 2013).

A number of quantitative wood anatomical traits useful for parameterizing hydraulic architecture have been proposed (Carlquist, 2001; Scholz, Klepsch, Karimi, & Jansen, 2013; von Arx et al., 2017). For example, vessel size and densities per given sapwood area define the amount of water that may be conducted through stems, but large vessels increase risk of vessel cavitation under drought events (Tyree & Zimmermann, 2002). Other characters such as the spatial distribution of vessels within the sapwood can have bearing on hydraulic efficiency and safety (Lens et al., 2011; Scholz et al., 2013). The connectivity of vessels for instance may increase the efficiency of water transportation, but may also enable gas embolisms to spread, leading to more dysfunctional vessels during drought conditions (Lens et al., 2011; Loepfe, Martinez‐Vilalta, Piñol, & Mencuccini, 2007).

Vessel‐associated tissues can also play important auxiliary roles in plant water relations. Parenchyma tissues in the xylem are a collection of living cells which can store water, contribute to lateral water transport, and also play important roles in refilling embolisms in vessels (de Micco, Balzano, & Wheeler, 2016; Morris et al., 2016, 2018 ; Poorter et al., 2010). In times of water deficit, vessels can become occluded by outgrowths of parenchyma or other biological material (Kitin et al., 2010; Schuldt et al., 2011). These occlusions in turn help to maintain the positive pressures in plant stems required for refilling embolized vessels (Canny, 1997, 1998 ).

In addition to wood traits, leaf traits may also respond to environmental stress, given that leaves are primary organs for plant photosynthesis and transpiration. During drought, plants commonly shed leaves, produce smaller, thinner, or “cheaper” leaves (i.e., leaves with lower leaf mass per area; Peña‐Rojas, Aranda, Joffre, & Fleck, 2005; Wolfe, Sperry, & Kursar, 2016), or reducing biomass partitioning to leaves (van Hees, 1997). Plants also have varying capacities to modulate their leaf water status (leaf water potentials; Ѱ), reflecting their ability to tolerate drought (Mitchell et al., 2013; Rowland, Costa, et al., 2015).

Although the aforementioned functional traits have been investigated under various contexts, there have been limited studies on how mature tropical trees in situ will respond in their functional anatomy to drought (Anderegg et al., 2013; Schuldt et al., 2011). Our aim was to address this knowledge gap by studying trees in a rainfall exclusion experiment in a tropical forest. We hypothesized therefore that trees can modify their wood functional anatomy and leaf traits to be hydraulically safer in response to soil moisture deficit. We predicted that relative to control individuals, the stem wood of drought‐affected trees will have smaller, less numerous, and more solitary vessels, a higher frequency of occluded vessel, a reduced proportion of storage tissue, and higher wood density. For leaf traits, we predicted that drought‐affected trees will have more negative leaf water potentials, lower ratios of leaf mass relative to branch mass and leaf areas to conducting sapwood area, higher leaf mass per unit area and leaf dry matter content, lower leaf areas, and thinner leaves.

## 2 MATERIALS AND METHODS

### 2.1 Study site and experimental drought setup

Our experiment is located within a tropical lowland rainforest within the Daintree Rainforest Observatory, Cape Tribulation, northeastern Australia (16°06′20′′S 145°26′40′′E, 50 m a.s.l.; Tng et al., 2016). The site experiences mean temperatures of 24.4°C and a high annual average rainfall of 4,900 mm/annum, the bulk of which falls during the wet season between October and February (Bureau of Meteorology, 2015). The forest occurs over acidic, dystrophic, and rocky soils, with canopy heights ranging from 24 to 47 m (Tng et al., 2016).

The in situ drought experiment was established in May 2015 and situated within a 1‐ha long‐term forest monitoring plot with canopy crane access (detailed in Laurance, 2015). The infrastructure to exclude rainfall consists of two rectangular 0.2 ha patches (i.e., 4,000 m^2^), with the remaining 0.6 ha of the plot serving as a control experimental patch (henceforth referred to as the drought and control treatments). At each of the two rectangular 0.2‐ha drought patches, clear‐panel roofing structures were installed in a tapering fashion between rows of aluminum troughs used for channeling rainwater away. Slits were cut in the roofing panel to enable plants tree stems and crowns above the roofing panels (tapering at 2.8 m height). Leaf litter accumulating on the roofing panels or in the troughs was transferred back to the soil surface.

We obtained volumetric soil water content from soil moisture sensors installed at eight soil pits stratified across both control (four pits) and drought (four pits) treatments. Within each soil pit, volumetric soil water content was measured continuously using time domain reflectometry probes (CS616, Campbell Scientific, UK) installed to log soil moisture at four soil depths: 10, 50, 100, and 150 cm.

### 2.2 Study species

For our study, we sampled four tree species belonging to four different families, *Argyrodendron peralatum* (F.M.Bailey) Edlin ex J.H.Boas (Malvaceae), *Endiandra microneura* C.T.White (Lauraceae), *Myristica globosa* (Warb.) W.J.de Wilde (Myristicaceae), and *Syzygium graveolens* (F.M.Bailey) Craven & Biffin (Myrtaceae) (Supporting Information Table S1). These species are typical of mature phase tropical rainforests and were chosen because of their high relative abundance within the study plot (Supporting Information Table S1; Tng et al., 2016).

We sampled plants 24 months after rainfall exclusion, in June 2017. Sampled trees were mature individuals of roughly similar‐sized diameters—three are canopy species and one midstorey (*Myristica*) although all canopies were well‐illuminated and largely unsuppressed by lianas or adjacent tree crowns (Supporting Information Table S1). We accessed tree canopies with the canopy crane, collecting branches from 37 trees representing 6–12 individuals of each study species (Supporting Information Table S1). While constrained by the availability of suitable trees to sample within each treatment, we endeavored to collect from at least four individuals of each species in each treatment, with the exception of *Endiandra*, for which only two drought‐affected individuals were accessible.

### 2.3 Wood traits

We collected one fully illuminated 50‐cm‐long branch from each individual from which we sampled two 5‐cm‐long branch segments for wood density following a standard protocol (Perez‐Harguindeguy et al., 2013). For this purpose, we split the branch segment lengthwise and removed pith before obtaining wood volumes. To standardize samples within and across species for anatomical measurements, we trimmed this branch to a length of 30 cm from the shoot tip and used the distal end (cross‐sectional diameters ranged between 5 and 8 mm) of this shorter branch for anatomy. We sampled an additional four to five 30‐cm‐long branches for leaf traits (See next section).

To examine differences in functional anatomy between control and drought‐affected trees, we measured a set of wood anatomical traits related to water transport (Scholz et al., 2013). Sampling from the distal ends of the 30‐cm‐long branches, we prepared transverse microscopic sections using a GSL1 portable microtome (Gärtner, Lucchinetti, & Schweingruber, 2014). Sections were stained with Toluidine blue and mounted onto microscope slides with glycerin jelly for examination.

We restricted our vessel sampling to the most recent sapwood growth in the outermost two growth rings. Using a digital camera (Nikon DS‐Fi2) mounted on a light microscope (Nikon ECLIPSE Ci‐L), we captured four digital microphotographs, one at each designated cardinal point of the branch transverse section. The digital images were processed in imaging software GIMP (v2.8.10), using the paintbrush function to color in the hollow lumen areas of 60–200 vessels in total from the four microphotographs per individual. We then used the imaging software Image J to measure vessel lumen area from the colored vessel lumens and to obtain mean and maximum vessel lumen areas (VA_mean_ and VA_max_, μm^2^). Vessel density (vessels/mm^2^) was measured by subsampling and counted colored vessels within a digital square frame of a 215,000 μm^2^, and vessel fraction (%) calculated as the percentage of area within our sampling frame occupied by vessels. The Hagen–Poiseuille equation (Tyree & Zimmermann, 2002) was used to calculate the stem theoretical conductivity(K_s_, Kg m^‐1^ MPa^‐1^ s^‐1^) per unit of xylem cross‐sectional area as *K*_s_ = π Σ*d* ^4^/128ƞA_cross‐sectional area_, where ƞ is the viscosity (1.002 × 10^‐9^ MPa s^‐1^) of water at 20°C.

To estimate the relative proportions of different cell types within our square sampling frame, we imposed a grid with 25 intersections in each frame (100 intersections total) and classified the cell types at each intersection into (a) vessels (including vessel cell lumens and walls), (b) parenchyma (both ray and axial), and (c) fibers (fiber cell lumens and walls). This grid‐sampled vessel fraction (relative vessel fraction) is relative to other cell fractions and distinguished from the earlier vessel lumen fraction which is based on sampling absolute vessel lumen areas. The two variables are strongly correlated (Pearson correlation: *r* = 0.86, *p* < 0.0001), but we report both for continuity.

Finally, as vessel spatial distribution or occlusions in vessels may affect hydraulic efficiency, we measured two additional vessel attributes. We obtained a vessel grouping index (unitless) using a larger sampling of 120 vessels examined throughout the outermost ring of the branch sapwood (including also the vessels sampled for VA). The vessel grouping index is the total number of vessels divided by the number of groups of vessels (i.e., vessels in direct contact with one another) (Scholz et al., 2013), and thus, a high value denotes a high vessel connectivity while values approximating 1 denote a tendency toward solitary vessels. To estimate the percentage of vessels occluded, we counted as occluded all tylosed or partially tylosed vessels, or vessels with infillings by gums or tannins, and expressed this as a percentage of the full complement of vessels examined.

### 2.4 Other branch‐level and leaf traits

We used the 30‐cm branch samples to estimate branch‐level twig dry matter content (mg/g) and leaf dry mass relative to twig dry mass (unitless). For this purpose, we weighed the fresh masses of the twigs and also the dry masses of the leaves and twigs after drying at 70⁰C for 48 hr. We also measured a standard set of functional leaf traits (Perez‐Harguindeguy et al., 2013) including leaf area (mm^2^), leaf mass per unit area (mg/mm^2^), and leaf dry matter content (mg/g). For leaf lamina thickness (mm), we measured using an electronic micrometer five leaves per individual at the mid‐leaf, taking care to avoid secondary veins. Additionally, we used the leaf areas calculated from our sampled branches to obtain the leaf area‐to‐sapwood area ratio (m^2^/mm^2^), which describes the relationship between projected leaf area and the area of sapwood supplying water to the leaves (Perez‐Harguindeguy et al., 2013).

As an indicator of plant water status, we measured pre‐dawn (Ѱ_pre‐dawn_) and mid‐day (Ѱ_mid‐day_) leaf water potentials (MPa) on two leaves from each individual using a pressure chamber (Model 1000; PMS Instruments, Corvallis, OR, USA).

### 2.5 Data analyses

To provide a context of the degree of soil water deficit within the rainfall exclusion experiment, we examined soil water differences between drought and control areas with analysis of variance, with pit depth nested within the experiment treatment. Data were averaged per month for each depth at each pit over a two‐year period beginning 1st of May 2015.

As our overarching purpose was to examine whether the rainfall exclusion treatment led to changes in traits across and within species, we fitted linear mixed effect models with wood or leaf traits as response variables, treatment (control or drought) and species and their interactions as fixed effects, and species nested within plant family as a random effect. Models were fitted with the restricted maximum likelihood estimation, following a standard protocol of data exploration (Zuur, Ieno, & Elphick, 2010). Mean and maximum vessel areas, vessel density, theoretical conductivity, leaf area, and leaf mass per unit area were log‐transformed, and the relative vessel fraction and percentage of vessels occluded were arcsine square root transformated before analysis. Because we were interested to examine how treatment affected traits within species, we used post hoc Tukey tests to determine whether treatment was a significant predictor of individual within‐species trait variation.

All analyses were performed in R (R Core Team, 2018). Linear mixed effect models were fitted using the lmer() function in the *lme4* package, and we used the ANOVA() function in the *car* package to generate ANOVA tables for these lmer model objects (Fox & Weisberg, 2011). For ease of interpretation of model results, we report these ANOVA tables, but present the full linear mixed effects models results in the Supplementary Material (Supporting Information Table S2). We examined differences within species as a result of treatment with Tukey post hoc tests (α = 0.05) using the *multcomp* package.

Because vessel areas represent a key hydraulic trait, we were interested also to examine the variation in vessel sizes to visualize hydraulic safety‐efficiency mechanisms (Tyree & Zimmermann, 2002). We therefore constructed vessel area class distributions for each species using the full complement of vessels (60–200 vessels) colored digitally following Apgaua et al. (2017). To accomplish this, we first sorted into proportions (%) the vessels contributing to each vessel area class, using 19 vessel area classes of 500‐μm^2^ intervals (maximum vessel area class between 9,000 and 9,500 μm^2^). We then averaged the vessel areas of each class across all individuals in each treatment and calculated the percentage frequency of vessels within each class.

To summarize the overall pattern of trait change and to detect trait co‐ordinations across species, we performed a principal components analysis (PCA) ordination of the studied individuals in trait space. For this purpose, we used only traits that were significant between treatments for at least one species. To improve ordination performance, trait values were standardized to 0–1 before ordination.

## 3 RESULTS

### 3.1 Soil water deficit

Over the two‐year period, the drought experiment succeeded in significantly drying the soils compared to the control treatment (ANOVA *F*_1,164 = _65.87, *p* < 0.0001). Soils in the drought experiment were 34.2% drier at the surface (10 cm), 25.9% drier at the subsurface (50 cm), and 14.3% and 9% drier at 100 and 150 cm depths, respectively (Supporting Information Figure S1). This interaction between drought treatment and depth was significant at surface and subsurface depths (ANOVA *F*_6,164 = _3.94, *p* = 0.001), illustrating the experiment successfully dried the surface soils but that lateral water movement was still apparent at greater depths across the site.

### 3.2 Overall and species‐specific responses to drought

**Figure 1:**
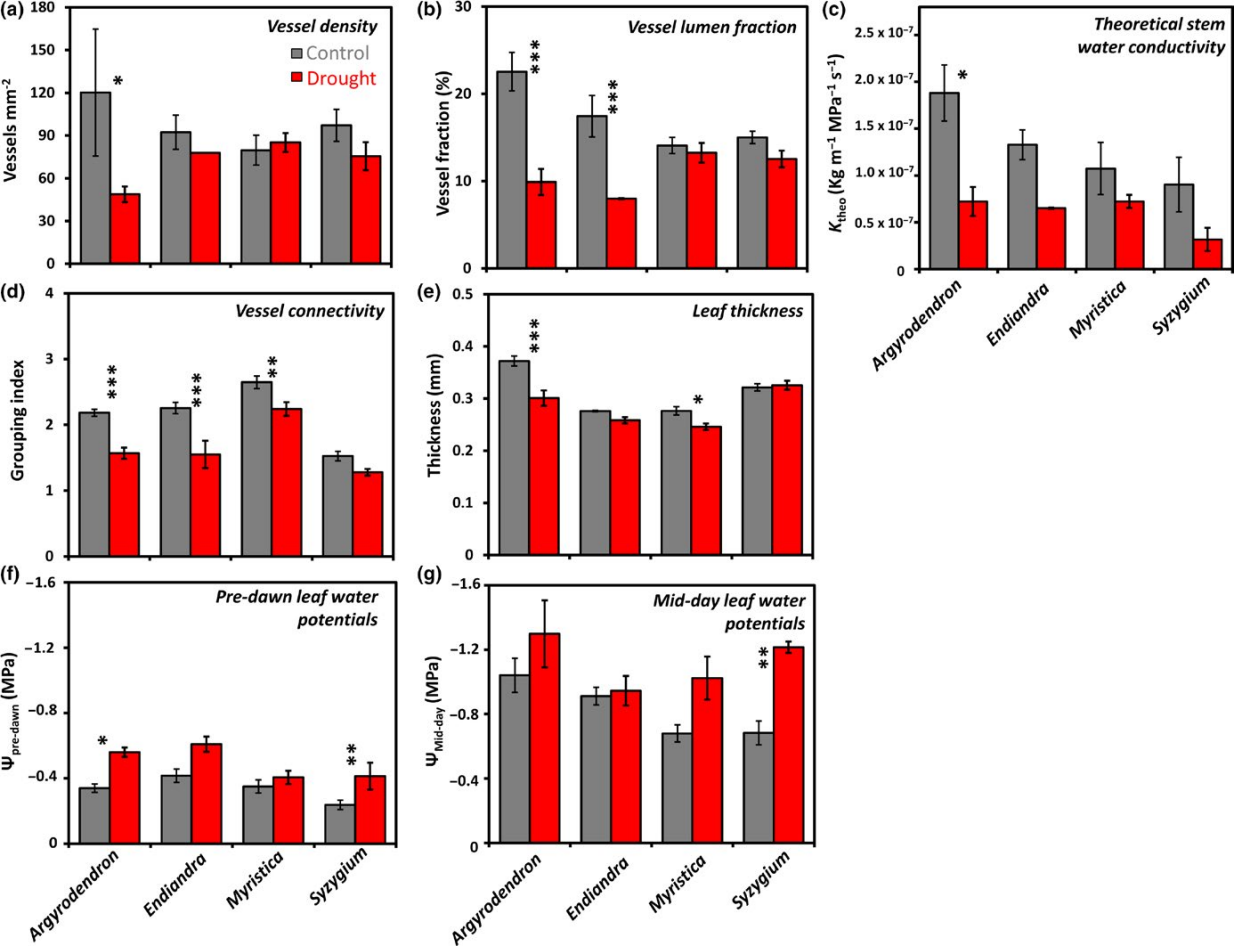
Plant trait means (±*SE*) of four tree species of lowland tropical rainforest trees in a rainfall exclusion experiment in a tropical lowland rainforest, Cape Tribulation, Australia. Species were replicated within a control and drought treatment areas (*Argyrodendron*: 4, 4; *Endiandra*: 4, 2; *Myristica*: 6, 6; *Syzygium*: 7, 4 individuals, respectively). Branch wood traits include (a) vessel density; (b) vessel fraction; (c) theoretical stem water conductivity; and (d) vessel connectivity (vessel grouping index). Leaf‐related traits include (e) leaf thickness, and (f) pre‐dawn and (g) mid‐day leaf water potentials. Only traits with significant responses within species are featured, based on post hoc Tukey test comparisons (*p* < 0.05*, <0.01**, <0.001***). See Table 1 for means (±*SD*) of all traits

We found significant effects of the drought experiment on all studied species for the wood anatomy and leaf‐related traits examined (Tables 1 and 2). Our linear mixed models demonstrate that the drought treatment alone was a significant predictor of lower vessel density and parenchyma fractions; increased fiber fractions and percentage of vessels occluded; and more negative pre‐dawn and mid‐day leaf water potentials. The drought treatment and species interactions also significantly explained lower vessel lumen fractions, relative vessel fractions, theoretical conductivity, vessel grouping index, relative vessel fraction, and leaf thickness (Figure 1; Table 2; Supporting Information Table S2).

**Table 1:** Means (±*SD*) for all wood and leaf‐related traits measured from the studied tree species under a throughfall exclusion experiment in a lowland tropical rainforest at the Daintree Rainforest Observatory, Cape Tribulation, Australia. Significantly different comparisons within species between the control and drought treatment based on post hoc Tukey tests are in bold

	*Argyrodendron peralatum*		*Endiandra microneura*		*Myristica globosa*		*Syzygium graveolens*	
	Control	Drought	Control	Drought	Control	Drought	Control	Drought
Wood traits								
Wood density (g/cm^3^)	0.563 ± 0.054	0.542 ± 0.004	0.580 ± 0.056	0.596 ± 0.078	0.46 ± 0.072	0.562 ± 0.105	0.564 ± 0.109	0.537 ± 0.053
Vessel lumen area (mean; μm^2^)	2292.143 ± 1067.741	1901.687 ± 335.995	1997.573 ± 754.447	1118.490 ± 157.821	1889.491 ± 812.411	1458.192 ± 191.717	1495.963 ± 405.603	1665.562 ± 347.841
Vessel lumen area (max; μm^2^)	5615.119 ± 2866.253	5348.555 ± 1039.822	4565.847 ± 955.716	2524.464 ± 559.890	4575.842 ± 1252.682	3964.737 ± 859.118	3531.129 ± 642.037	3448.954 ± 743.502
Vessel density (vessels/mm^2^)	**120.151 ± 89.046**	**48.757 ± 10.931**	92.290 ± 24.063	71.394 ± 7.373	79.714 ± 25.736	85.131 ± 16.263	100.532 ± 35.503	79.907 ± 17.084
Vessel lumen fraction (%)	**0.225 ± 0.044**	**0.099 ± 0.03**	**0.174 ± 0.047**	**0.078 ± 0.005**	0.141 ± 0.023	0.132 ± 0.028	0.142 ± 0.007	0.14 ± 0.030
Stem theoretical conductivity (Kg m^−^^1^ MPa^−1^ s^−^^1^)	**1.880 × 10^7^ ± 5.981 × 10^6^**	**7.221 × 10^6^ ± 3.085 × 10^6^**	1.328 × 10^7^ ± 6.507 × 10^6^	3.392 × 10^6^ ± 4.955 × 10^5^	1.073 × 10^7^ ± 6821652.5	7.231 × 10^6^ ± 1.723 × 10^6^	7.972 × 10^6^ ± 1.979 × 10^6^	9.048 × 10^6^ ± 3.582 × 10^6^
Vessels occluded (%)	0.208 ± 0.417	3.4 ± 3.339	0.602 ± 0.404	2.835 ± 2.43	**7.553 ± 5.179**	**26.107 ± 17.685**	0 ± 0	0.833 ± 1.394
Vessel grouping index (unitless)	**2.183 ± 0.104**	**1.568 ± 0.17**	**2.253 ± 0.173**	**1.483 ± 0.287**	**2.646 ± 0.234**	**2.24 ± 0.253**	1.598 ± 0.16	1.299 ± 0.108
Relative vessel fraction (%)	**29.5 ± 9.539**	**15.5 ± 3.873**	23.75 ± 5.315	12 ± 1.633	19.333 ± 4.131	17.5 ± 4.183	19.6 ± 4.827	21.333 ± 5.046
Relative parenchyma fraction (%)	49 ± 8.602	45.25 ± 4.272	40.5 ± 12.124	26.5 ± 1.291	**42.667 ± 8.335**	**26.833 ± 4.446**	**52.571 ± 1.343**	**35.25 ± 2.016**
Relative fiber fraction (%)	**21.5 ± 4.041**	**39.25 ± 3.5**	**35.75 ± 7.5**	**64 ± 7.528**	**38 ± 7.043**	**55.667 ± 3.724**	**25.571 ± 2.852**	**46.5 ± 2.630**
Leaf‐related traits								
Twig dry matter content (mg/g)	417.764 ± 18.568	406.455 ± 13.352	421.143 ± 98.014	414.545 ± 67.953	337.426 ± 17.803	361.456 ± 21.739	389.739 ± 68.353	388.279 ± 46.335
Leaf dry mass: twig dry mass (unitless)	4.057 ± 0.512	3.139 ± 1.386	3.458 ± 0.48	3.636 ± 0.175	3.921 ± 0.827	2.686 ± 1.035	3.1 ± 0.526	3.004 ± 0.505
Leaf area (cm^2^)	30.257 ± 6.754	24.501 ± 13.783	41.273 ± 2.782	35.258 ± 9.482	62.718 ± 21.109	54.704 ± 16.556	43.345 ± 16.484	61.341 ± 24.218
LMA (mg/mm^2^)	0.189 ± 0.012	0.193 ± 0.025	0.144 ± 0.022	0.143 ± 0.018	0.124 ± 0.020	0.122 ± 0.016	0.145 ± 0.023	0.138 ± 0.023
LDMC (mg/mg)	656.459 ± 226.726	557.63 ± 33.394	456.013 ± 99.955	567.675 ± 293.489	396.319 ± 35.96	386.414 ± 43.655	444.387 ± 78.838	504.544 ± 257.134
Leaf thickness (mm)	**0.372 ± 0.019**	**0.301 ± 0.03**	0.276 ± 0.003	0.244 ± 0.017	**0.277 ± 0.019**	**0.244 ± 0.016**	0.315 ± 0.015	0.33 ± 0.017
Ѱ_pre‐dawn_ (MPa)	**−0.340 ± −0.051**	**−0.540 ± −0.020**	−0.416 ± −0.081	−0.595 ± −0.040	−0.350 ± −0.098	−0.406 ± −0.010	**−0.205 ± −0.075**	**−0.414 ± −0.164**
Ѱ_mid‐day_ (MPa)	−1.041 ± 0.211	−1.199 ± 0.240	−0.786 ± 0.240	−1.339 ± 0.479	−0.596 ± 0.141	−1.023 ± 0.326	**−0.618 ± 0.121**	**−1.093 ± 0.241**
Leaf area: sapwood area (m^2^/mm^2^)	0.107 ± 0.042	0.095 ± 0.06	0.067 ± 0.031	0.067 ± 0.018	0.071 ± 0.026	0.078 ± 0.012	0.067 ± 0.027	0.058 ± 0.014

**Table 2:** Statistics of ANOVA tables calculated from linear mixed effects models (see Methods) fitting wood and leaf trait as responses, treatment (control vs. drought) and species as fixed effects, and species nested within family as a random effect. Significant p‐values are indicated by asterisks as follows: *p* < 0.05*, <0.01**, <0.001***. Degrees of freedom for treatment, species, and interactions are 1, 3, and 3, respectively. Full parameter estimates for linear mixed effects models are given in the Supporting Information Table S2

	Treatment		Species		Treatment × Species	
	Chi‐square	P	Chi‐square	P	Chi‐square	P
Wood density	11.514	0.283	11.744	0.759	44.240	0.219
Vessel area (mean)	20.909	0.148	13.452	0.718	35.171	0.319
Vessel area (max)	23.313	0.127	12.407	0.743	63.800	0.095
Vessel density	42.781	0.039*	0.835	0.841	77.760	0.051
Vessel lumen fraction	347.321	>0.001***	20.472	0.563	274.169	>0.001***
Theoretical conductivity	141.422	>0.001***	22.140	0.529	77.932	0.050*
Relative vessel fraction	146.472	>0.001***	14.603	0.692	88.362	0.032*
Relative parenchyma fraction	351.406	>0.001***	17.922	0.617	54.780	0.140
Relative fiber fraction	1.015.961	>0.001***	27.520	0.432	21.085	0.550
Vessel grouping index	514.795	>0.001***	46.204	0.202	85.801	0.035*
% vessels occluded	148.518	>0.001***	19.166	0.590	55.964	0.133
Twig dry matter content	54.015	0.020*	13.405	0.72	44.506	0.217
Leaf dry mass: twig dry mass	0.026	0.872	0.9014	0.825	16.049	0.658
Leaf area	0.004	0.951	0.7594	0.859	64.429	0.092
LMA	0.151	0.698	28.467	0.416	0.4775	0.924
Leaf dry matter content	0.165	0.685	12.773	0.735	0.394	0.941
Leaf thickness	20.368	>0.001***	6.041	0.110	19.252	>0.001***
Leaf area: sapwood area	0	0.996	12.209	0.748	11.683	0.761
Pre‐dawn leaf water potential	216.944	>0.001***	20.885	0.554	50.482	0.168
Mid‐day leaf water potential	180.664	>0.001***	16.730	0.643	42.994	0.231

Each species exhibited unique combinations of trait variations between individuals in the drought and control treatments, as our post hoc Tukey tests reveal (Figure 1). For instance, drought‐affected *Argyrodendron* had significantly lower vessel densities, vessel lumen fractions, stem theoretical conductivities, and vessel connectivity (Figure 1a‐d). Yet, within *Myristica* and particularly *Syzygium*, many of these traits did not vary between drought‐affected and control individuals. In *Endiandra*, the means of most of these traits were lower (or more negative in pre‐dawn leaf water potential) in drought‐affected individuals, although only significantly so in vessel lumen fraction and vessel connectivity.

**Figure 2:**
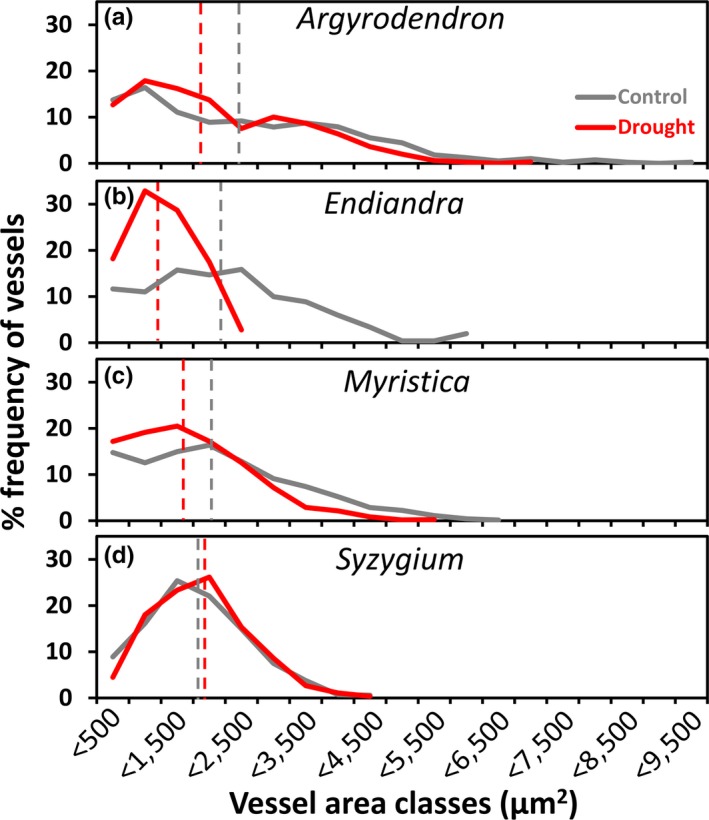
Vessel area class distribution within canopy branch sapwood for four species in a rainfall exclusion experiment in a tropical lowland rainforest, Cape Tribulation, Australia. Species are as follows: (a), *Argyrodendron peralatum*; (b), *Endiandra microneura*; (c), *Myristica globosa*; (d), *Syzygium graveolens* and replication follows Figure 1. The median vessel area classes of the three control and drought‐affected individuals are shown as dashed gray and red lines, respectively

A special note with regard to vessel sizes was that the average and maximum vessel lumen areas in drought‐affected *Endiandra* individuals appeared to be smaller compared to controls (Table 1), even though this difference was not significant. However, vessel area frequency distributions (Figure 2) corroborated this pattern, showing that drought‐affected individuals of *Endiandra* have fewer wide vessels and more narrow vessels.

**Figure 3:**
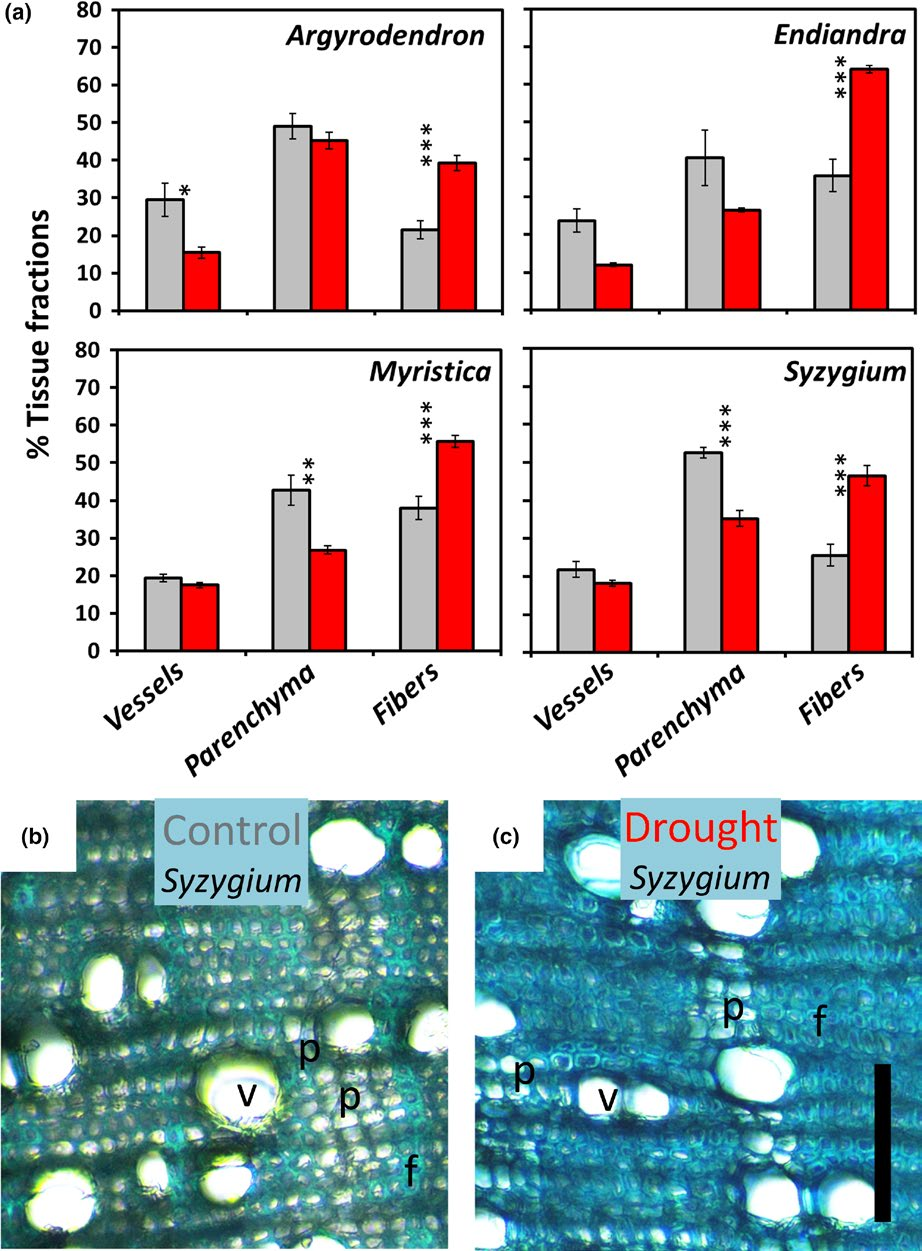
Tissue proportions within branch sections of trees from tropical lowland rainforest, Cape Tribulation, Australia. (a) Mean (±*SE*) proportions of vessels, parenchyma, and fibers from replicated individuals of the four study species within the control and drought treatment (replication as per Figure 1). Asterisks in parenthesis denote Tukey's test significance between control and drought‐affected individuals within each species (*p* < 0.05*, *p* < 0.01**, *p* < 0.001***). An example branch cross sections of (b) a control and (c) a drought‐affected individual of *Syzygium* illustrates the relative stem tissue fractions of vessels (v), storage parenchyma (p), and fibers (f)

Drought‐affected *Myristica* and *Syzygium* exhibited lower parenchyma tissue fractions than in their controls (Table 1, Figure 3a‐c), but these changes were not significant in *Argyrodendron* and *Endiandra*. Conspicuously, however, all species showed significantly higher fiber fractions in their drought‐affected individuals (Table 1, Figure 3a).

**Figure 4:**
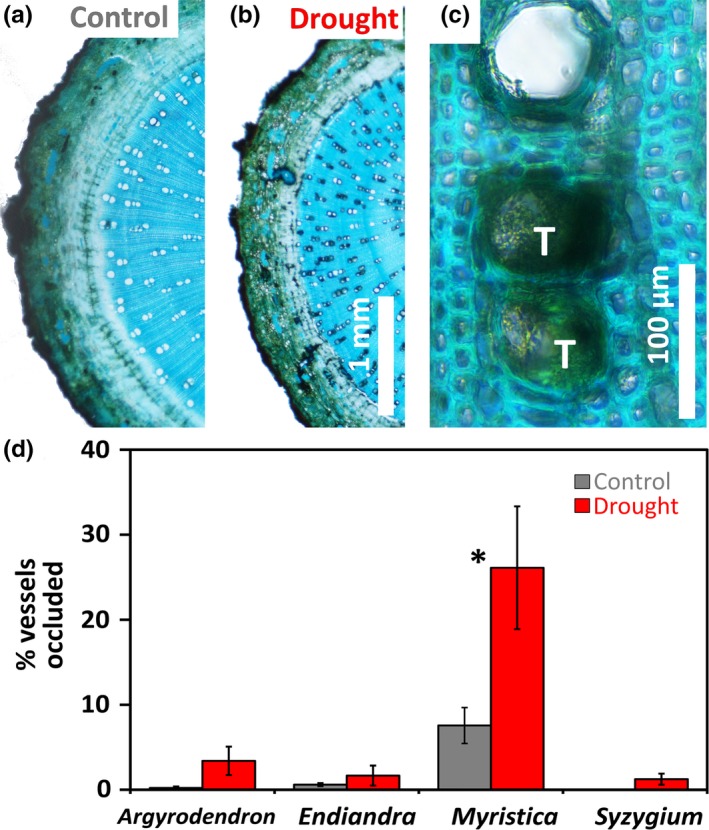
Drought‐induced vessel occlusions in tree branch cross sections. (a) Branch cross sections of *Myristica globosa* (replication as per Figure 1) demonstrate a lower frequency of vessel occlusion in control individuals of *Myristica* than in (b) drought‐affected individuals in a rainfall exclusion experiment in a tropical lowland rainforest, Cape Tribulation, Australia. At higher magnification (c), the occlusions are discernable as dark tyloses (white “T”s). All study species exhibited some degree of vessel occlusions (d). Asterisks denote significant results of Tukey's post hoc comparisons between control and drought‐affected individuals within each species (*p* < 0.05*, *p* < 0.01**, *p* < 0.001***)

Occluded vessels with biological material (tyloses or gums/tannins) were conspicuous in *Myristica* in particular (Figure 4a–c), where they occurred four times more frequently in the drought‐affected individuals (Table 1).

In terms of leaf‐related traits, the mean leaf lamina thickness was higher in control individuals of *Argyrodendron* and *Myristica*, but not significantly different in *Endiandra* and *Syzygium* (Table 1; Figure 1e). The pre‐dawn leaf water potentials were significantly more negative in drought‐affected individuals of *Argyrodendron*, and *Syzygium* (Table 1), but not in *Endiandra* and *Myristica* (Table 1; Figure 1f). Mid‐day leaf water potentials (Figure 1g) were significantly more negative in only drought‐affected individuals of *Syzygium* (Table 1).

### 3.3 Coordination of plant traits

**Figure 5:**
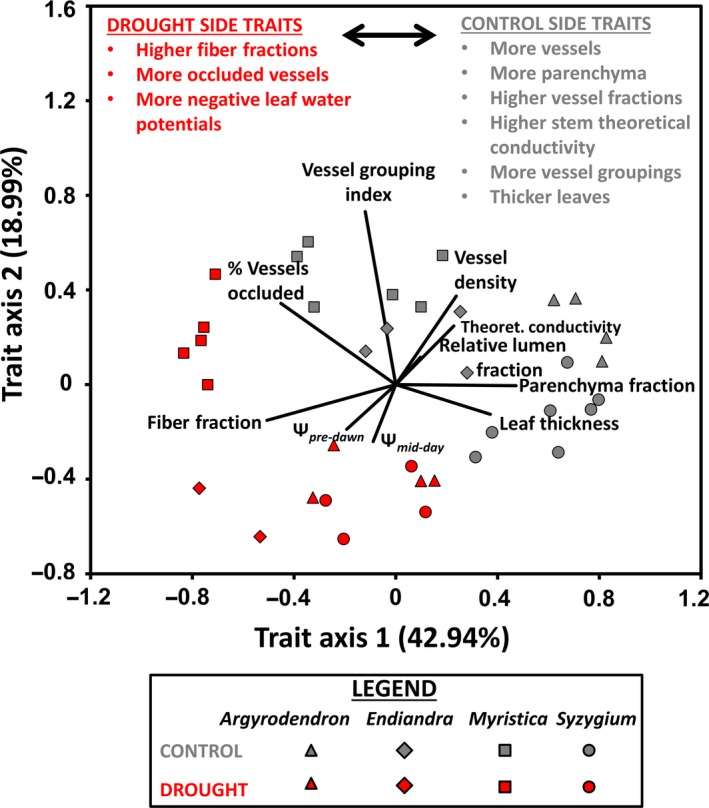
Summary of directional trait acclimation of all studied trees in trait space (principal components analysis axes) in a rainfall exclusion experiment in a tropical lowland rainforest, Cape Tribulation, Australia. The length of the vectors (dark lines radiating from the center) reflects the strength of the influence of individual variables on the overall variance of the sample set. Only traits with significant differences between control and drought‐affected individuals were used for the ordination (See Table 2), and vessel lumen fraction was not included in the analysis due to intercorrelation with relative vessel fraction. The first three axes accounted for 74.63% of the trait variation across all individuals (only first two axes shown). The control and drought‐affected individuals show segregation of trait composition in their wood and leaf traits, and also some species‐specific responses driven chiefly by single traits (e.g., high vessel occlusion by drought‐affected individuals of *Myristica globosa*) (for axis loadings, see Supporting Information Table S3)

An principal components ordination analysis of individual trees in trait space shows a coordinated shift in trait profiles (Figure 5), where drought‐affected individuals clustered on one side of the ordination space, driven by high fiber values, vessel occlusion, and negative leaf water potentials, while control individuals on the other side, driven by higher numbers of vessel grouping, larger maximum vessel areas, higher theoretical conductivities, higher parenchyma fractions, and thicker leaves (Figure 5; Supporting Information Table S3).

We also found that the leaf traits (pre‐dawn and mid‐day leaf water potentials, and leaf thickness) were mostly negatively connected with vessel traits. However, leaf thickness and parenchyma fraction were coordinated, as were leaf water potentials with wood fiber fractions (Figure 5).

## 4 DISCUSSION

A biophysical basis of drought adaptation is fundamental for understanding how recurring drought will affect tropical forests. Examining a broad suite of wood and leaf trait responses of tropical rainforest trees to a two‐year in situ rainfall exclusion experiment, we found clear evidence of intraspecific changes in wood and leaf traits consistent with hydraulic architecture changes in situ under short‐term water deficit. The key advantage of our study is replication within each species, allowing us to verify that individual variation is not simply idiosyncratic, but reflects species‐specific responses to drought.

### 4.1 Vessel areas, density, and theoretical conductivity

We observed in one species, *Endiandra*, that drought‐affected individuals had smaller mean and maximum vessel areas than control individuals, although the mean of these traits was not significant different. However, detecting significant change in vessel area is challenging because this trait can vary greatly within and between individual trees (Apgaua et al., 2015). Hence, the interpretation of this feature may benefit by visualizing vessel area class distributions rather than simply averaging a range of values (e.g., Apgaua et al., 2017), which corroborate that in *Endiandra*, control individuals had a wider range of vessel areas and a higher proportion of vessels in the larger area classes than drought‐affected individuals.

The higher vessel fractions and theoretical conductivities in control individuals of *Argyrodendron* corroborate with data from a rainfall exclusion experiment in Sulawesi, where Schuldt et al. (2011) found higher sapwood‐specific water conductivity in control individuals of their study species *Castanopsis acuminatissima*. The differences in theoretical conductivity in spite of the lack of difference in vessel dimensions and densities in *Argyrodendron* may be due to the way conductivity scales nonlinearly with small increments in vessel area (Tyree & Zimmermann, 2002). Therefore, even though the mean vessel areas and densities were not significantly different between the control and drought‐affected individuals of *Argyrodendron*, the means of these traits in the control individuals were visibly higher, and this was sufficient to scale consistently to higher vessel fractions and conductivity in these control trees. In *Myristica* and *Syzygium*, vessel areas, densities, fractions, and theoretical conductivities did not vary between treatments, possibly reflecting a genotypic constrain in plasticity in these traits.

### 4.2 Tissue fractions, vessel connectivity, and occlusions, and their implications

The intraspecific differences in relative tissue fractions reveal an additional dimension in adaptive functional anatomy, which, to the best of our knowledge, represents a novel observation in tropical trees. Parenchyma fractions within *Myristica* and *Syzygium* were significantly higher in the control individuals and may reflect an important water‐use strategy by these species. Specifically, the reduced parenchyma fractions of drought‐affected individuals of these species may be due to the use of stored water in the parenchyma. Indeed, plant vessels can become dysfunctional when embolisms develop in the water column during periods of water deficit, and the embolism refilling has often been documented in plant species that contain vessel‐associated parenchyma cells, emphasizing a tight linkage of living tissue and vessels (for reviews, see Brodersen & McElrone, 2013; Morris et al., 2016).

Counterbalancing the vessel and parenchyma fractions, we found consistently that fiber fractions were proportionally lower in the control individuals in all the study species. This seems to fit well within the scheme of plants adapting to water deficit as fibers may confer resistance to vessels against negative pressures during drought (Cai, Li, Zhang, Zhang, & Tyree, 2014). However, further research is needed to ascertain whether the increase in fiber portions in drought‐affected individuals is due to a higher investment in these tissues or a passive consequence of other cell fractions decreasing in tandem.

In addition to tissue fractions, the formation of vessel occlusions appears to vary across species, as *Myristica* exhibited a pronounced tendency for this feature which we did not observe to the same degree in the other study species. Schuldt et al. (2011) similarly found conspicuous occluded vessels in drought‐affected *Castanopsis* trees in their Sulawesi study, although they did not present quantitative data. Canny (1997) attributes the occlusion of vessels by tyloses as a response to embolisms during water deficit and suggests that these occlusions serve to preserve the tissue pressure which will later enable plants to expresses water to refill embolisms in remaining vessels. Tyloses, which are parenchyma tissues, render the embolized‐vessel gas space incompressible, thus minimizing stem pressure that may be otherwise dissipated by compressing the gas in such spaces (Canny, 1997, 1998).

Changes in vessel spatial arrangement were observed consistently across species. Chiefly, we report for the first time in an experimentally induced drought a tendency for species to have less vessel groups and hence lower vessel connectivity. How downregulating vessel grouping impacts water conduction is unclear, but theoretically, this should minimize the risk of embolism propagating to other vessels during drought conditions (Lens et al., 2011).

### 4.3 Leaf‐related traits

While the study has focused on functional wood anatomy, we also found instances of leaf‐level changes to water deficits. Firstly, our leaf water potential data establish that drought‐affected individuals of all our target species more water‐stressed than control individuals, as might be expected (Inoue et al., 2017). Our finding of thinner leaves in the drought‐affected individuals of all study species is consistent with the dehydrated status of the sampled leaves, in line with the findings of Binks et al. (2016a), who reported moderately thinner leaves and also thicker leaf underside epidermis in Amazonian trees subjected to experimental drought. However, we did not find evidence of decreasing leaf areas in our species, which has been reported more conclusively in the Sulawesi experiment (Schuldt et al., 2011).

### 4.4 Synthesis

We demonstrate that tropical trees respond in their hydraulic architecture traits to short‐term water deficit, representing diverse trait strategies across species (Apgaua et al., 2015). In both wood and leaf traits, species varied in their capacity to modify specific traits, as visualized in various trait trade‐offs or co‐ordinations when species were ordinated in trait space. We interpret these as unique hydraulic strategies that different species use to deal with short‐term drought.

While the study has not progressed as long as some other rainfall exclusion experiments (e.g., 15 years, Caxuiana, Brazil; Rowland, Costa, et al., 2015; Rowland, Lobo‐do‐Vale, et al., 2015), our study differs from the Caxuiana study which showed many morphological, anatomical, and physiological parameters that did not respond to drought, even after 15 years of rainfall exclusion. This difference may be due to the difference in traits and tree sampling (genus‐level sampling at Caxuiana), or fundamental ecological differences between the Australian and Brazilian systems. Nevertheless, we recommend that further studies examining drought responses should integrate anatomical traits such as cell fractions and indices of vessel connectivity with physiological measures and examine more closely the dynamics of wood and leaf trait couplings or associations.

Our findings open new avenues for understanding plant responses to environmental stress and suggest that forest stands may have hidden complexities with critical implications for forest functioning and response to disturbances. For instance, it is unclear whether these trait changes are active or passive responses, and whether different species or plant functional groups may modify some but not other traits actively. Our results suggest that scaling up from individual trees to tree stands can be fraught with risk and further illustrate that tropical tree species are not functionally equivalent with respect to their impacts on ecosystem processes. Understanding the limits to trait plasticity within species and genera is therefore important and will help with building and refining trait‐based predictive models of plant adaptation to drought. We also envision that incorporating plant hydraulic traits into terrestrial ecosystem and biosphere models will facilitate more robust predictions of how future climate change will impact tropical forests globally.

## CONFLICT OF INTERESTS

None declared.

## AUTHOR CONTRIBUTIONS

DYPT, DMGA, and SGWL conceived the study; DYPT, DMGA, and FYI executed the study and collected the data; DYPT, DMGA, and SGWL analyzed the data; DYPT led the writing of the manuscript. All authors contributed significantly to the drafts and gave final approval for publication.

## DATA ACCESSIBILITY

All relevant data are within the paper and its Supporting Information files.
