# Supplementary material for: Rainforest trees respond to drought by modifying their hydraulic architecture
Source: Ecol Evol. 2018 Dec 11;8(24):12479–91. doi: 10.1002/ece3.4601 (PMC6308889; doi:10.1002/ece3.4601)
Supplement: Supplementary file 1 [file ECE3-8-12479-s001.docx]

**Rainforest trees respond to drought by modifying their hydraulic architecture**

David Y.P. Tng^1,2*^, Deborah M.G. Apgaua^1^, Yoko F. Ishida^1^, Maurizio Mencuccini^3^, Jon Lloyd^1,4.5^, William F. Laurance^1^, and Susan G.W. Laurance^1^

^1^Centre for Tropical, Environmental and Sustainability Sciences, College of Science and Engineering, James Cook University, 14-88 McGregor Rd, Smithfield Qld 4878, Australia

^2^Instituto de Biologia, Universidade Federal da Bahia, R. Barão Jeremoabo, Ondina, 40170-115 Salvador, Bahia, Brazil

^3^School of GeoSciences, University of Edinburgh, Edinburgh EH9 3JN, UK

^4^Department of Life Sciences, Imperial College London, Silwood Park Campus, Ascot SL57PY, UK

^5^Faculdade de Filosofia, Ciencias e Letras de Ribeirao Preto, Universidade de Sao Paulo, 14040-900 Ribeirao Preto, Brazil

*e-mail: davetngcom@gmail.com


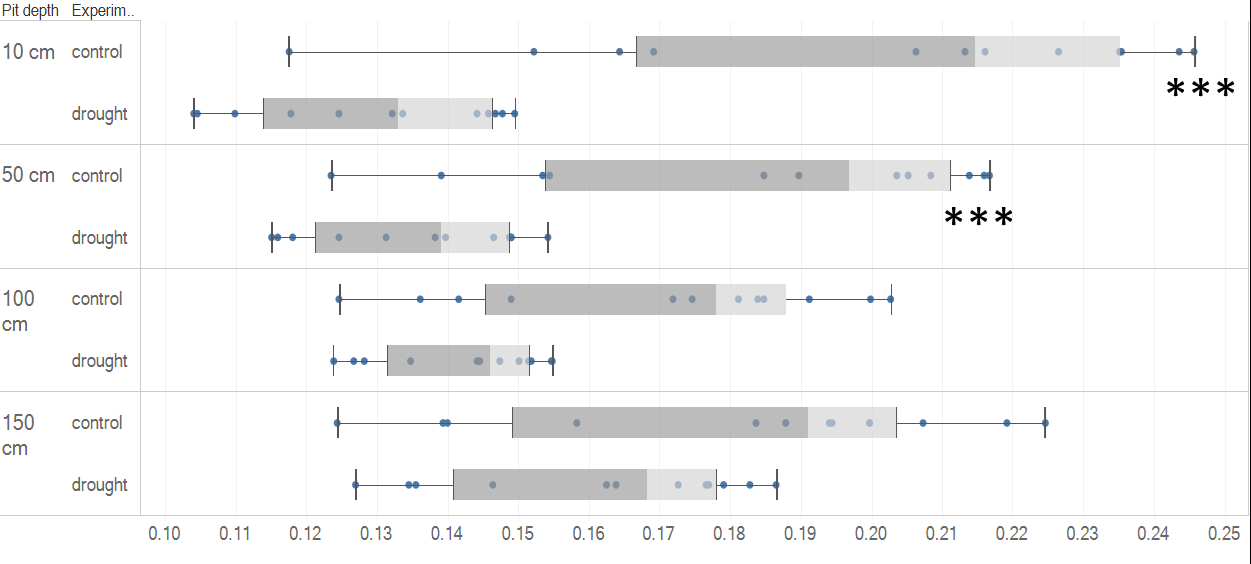


**Figure S1.** Volumetric soil water content of the control and the drought areas of the study plot during the 2-yr experimental period. The data is based on sensors installed in four soil pits each within the control and drought areas of the plot. Each box encompasses the 25th to 75th percentiles; the median is indicated by the meeting point between the dark grey and light grey portions of each boxplot and the other horizontal lines outside the box indicate the 10th and 90th percentiles. Dots indicate the averages of each calendar month between 2015 and 2017. One-way ANOVAs were performed on the data (log-transformed) and significant post-hoc p-values are indicated by asterisks as follows: *P*<0.05*, <0.01**, <0.001***.

**Table S1.** Targeted canopy tree species from a lowland tropical rainforest at the Daintree Rainforest Observatory, Cape Tribulation, Australia. Replication number (n), and the mean values (±SD) of stem diameters at breast height (DBH) and tree heights are indicated for trees within the Control and Drought treatment respectively.

| **Species** | **Family** | ***n* (control, drought)** | **DBH (cm)** |  | **Height (m)** |  |
| --- | --- | --- | --- | --- | --- | --- |
|  |  |  | **Control** | **Drought** | **Control** | **Drought** |
| *Argyrodendron peralatum* (F.M.Bailey) Edlin ex J.H.Boas | Malvaceae | 4, 4 | 46.6, ±14.3 | 32.0, ±4.7 | 31.6, ±3.3 | 27.2, ±5.7 |
| *Endiandra microneura* C.T.White | Lauraceae | 4, 2 | 31.2, ±6.3 | 44.6, ±0.8 | 24.3, ±2.1 | 25.8, ±2.1 |
| *Myristica globosa* (Warb.) W.J.de Wilde | Myristicaceae | 6, 6 | 28.6, ±8.7 | 30.0, ±6.6 | 20.1, ±3.9 | 19.1, ±3.1 |
| *Syzygium graveolens* (F.M.Bailey) Craven & Biffin | Myrtaceae | 7, 4 | 57.1, ±8.8 | 41.9, ±11.7 | 28.3, ±2.1 | 28.7, ±4.8 |

**Table S2.** All parameter estimates (±S.E) and random effect variances for linear mixed effects models fitted with a restricted maximum likelihood estimation for wood and leaf trait as responses, and treatment (Control vs. Drought) and Species as fixed effects, and Species nested within Family as random effects. Significant p-values are indicated by asterisks as follows: *P*<0.05*, <0.01**, <0.001***. Refer to Table 2 in the main text for anova tables of each model (See also Materials and Methods).

|  |  | Fixed effects: | |  |  |  |  |  |  | Random effect variances | | |
| --- | --- | --- | --- | --- | --- | --- | --- | --- | --- | --- | --- | --- |
|  |  | (Intercept) | Treatment-Drought | Species-*Endiandra* | Species-*Myristica* | Species-*Syzygium* | Treatment-Drought:Species-*Endiandra* | Treatment-Drought:Species-*Myristica* | Treatment-Drought:Species-*Syzygium* | Species nested in Family | Family | Residual |
| Wood density | Estimate | 0.563 | -0.020 | 0.017 | -0.102 | -0.007 | 0.038 | 0.122291 | 0.004 | 0.001 | 0.001 | 0.006 |
|  | Std err. | 0.058 | 0.054 | 0.083 | 0.079 | 0.079 | 0.086 | 0.070273 | 0.073 |  |  |  |
|  | T | 9624 | -0.377 | 0.202 | -1288 | -0.094 | 0.444 | 1740 | 0.055 |  |  |  |
|  | P | >0.001*** | 0.709 | 0.841 | 0.2079 | 0.9261 | 0.66 | 0.0924 | 0.956 |  |  |  |
| Vessel area (mean) | Estimate | 330834 | -0.035 | -0.032 | -0.063 | -0.126 | -0.233 | -0.049 | 0.054 | 0.002 | 0.002 | 0.022 |
|  | Std err. | 0.096 | 0.104 | 0.135 | 0.128 | 0.126 | 0.164 | 0.134 | 0.138 |  |  |  |
|  | T | 34595 | -0.337 | -0.236 | -0.494 | -0.999 | -1425 | -0.367 | 0.388 |  |  |  |
|  | P | >0.001*** | 0.739 | 0.815 | 0.625 | 0.326 | 0.165 | 0.716 | 0.701 |  |  |  |
| Vessel area (max) | Estimate | 369994 | 0.022 | -0.047 | -0.054 | -0.160 | -0.318 | -0.078 | -0.034 | 0.065 | 0.104 | 0.121 |
|  | Std err. | 0.137 | 0.086 | 0.194 | 0.191 | 0.190 | 0.135 | 0.111 | 0.114 |  |  |  |
|  | T | 26926 | 0.256 | -0.242 | -0.282 | -0.843 | -2348 | -0.709 | -0.297 |  |  |  |
|  | P | >0.001*** | 0.7999 | 0.8108 | 0.7798 | 0.4062 | 0.026* | 0.4842 | 0.769 |  |  |  |
| Vessel density | Estimate | 460700 | -0.739 | -0.112 | -0.270 | -0.063 | 0.599 | 0.832 | 0.489 | 0.08 | 0.08 | 0.329 |
|  | Std err. | 0.19978 | 0.233 | 0.283 | 0.266 | 0.261 | 0.368 | 0.300 | 0.311 |  |  |  |
|  | T | 23061 | -3178 | -0.398 | -1016 | -0.241 | 1628 | 2768 | 1571 |  |  |  |
|  | P | >0.001*** | 0.004 | 0.693 | 0.318 | 0.811 | 0.114 | 0.010** | 0.127 |  |  |  |
| Vessel lumen fraction | Estimate | -1505 | -841.8 | -267 | -466.6 | -398.7 | 85.17 | 773.2 | 659 | 0.001 | 0.001 | 0.04 |
|  | Std err. | 101.8 | 135.4 | 144 | 133 | 129.6 | 214.1 | 174.8 | 181 |  |  |  |
|  | T | -14775 | -6216 | -1854 | -3509 | -3076 | 0.398 | 4423 | 3642 |  |  |  |
|  | P | 0.999 | >0.001*** | 0.999 | 0.999 | 0.999 | 0.694 | >0.001*** | 0.001** |  |  |  |
| Stem theoretical conductivity | Estimate | 1.88E+10 | -1.16E+10 | -5.53E+09 | -8.07E+09 | -9.77E+09 | 1.49E+09 | 8.08E+09 | 1.03E+10 | 2.35E+15 | 2.35E+15 | 2.03E+16 |
|  | Std err. | 3.13E+09 | 3.19E+09 | 4.42E+09 | 4.23E+09 | 4.17E+09 | 5.04E+09 | 4.11E+09 | 4.26E+09 |  |  |  |
|  | T | 4.36E-06 | 2.32E+14 | 4.36E-06 | 3.64E-06 | 3.45E-06 | 9.99E+14 | 9.92E+14 | 4.99E+15 |  |  |  |
|  | P | 1.00E+06 | 2.78E-04 | 1.00E+06 | 1.00E+06 | 1.00E+06 | 7.68E-01 | 4.95E-02 | 1.60E-02 |  |  |  |
| Relative vessel fraction | Estimate | 0.570 | -0.168 | -0.063 | -0.117 | -0.087 | 0.015 | 0.143 | 0.126 | >0.001 | >0.001 | 0.004 |
|  | Std err. | 0.038 | 0.045 | 0.053 | 0.050 | 0.049 | 0.071 | 0.058 | 0.060 |  |  |  |
|  | T | 15123 | -3751 | -1190 | -2329 | -1770 | 0.207 | 2479 | 2100 |  |  |  |
|  | P | >0.001*** | >0.001*** | 0.244 | 0.027* | 0.087 | 0.837 | 0.019* | 0.045* |  |  |  |
| Relative parenchyma fraction | Estimate | 49000 | -3750 | -8500 | -6333 | 3571 | -10250 | -12083 | -13571 | 42.5 | 29.52 | 44.44 |
|  | Std err. | 9118 | 4714 | 12894 | 12750 | 12708 | 7454 | 6086 | 6299 |  |  |  |
|  | T | 5374 | -0.795 | -0.659 | -0.497 | 0.281 | -1375 | -1986 | -2154 |  |  |  |
|  | P | >0.001*** | 0.433 | 0.515 | 0.623 | 0.781 | 0.180 | 0.0566 | 0.040* |  |  |  |
| Relative fibre fraction | Estimate | 2150000 | 1775000 | 1425000 | 1650000 | 407143 | 800000 | -0.083 | 317857 | 45.93 | 27.74 | 34.38 |
|  | Std err. | 906987 | 414614 | 1282674 | 1271456 | 1268233 | 655562 | 535264 | 554051 |  |  |  |
|  | T | 2370 | 4281 | 1111 | 1298 | 0.321 | 1220 | -0.016 | 0.574 |  |  |  |
|  | P | 0.025 | >0.001*** | 0.276 | 0.205 | 0.750 | 0.232 | 0.988 | 0.571 |  |  |  |
| Vessel grouping index | Estimate | 218266 | -0.615 | 0.071 | 0.463 | -0.658 | -0.178 | 0.209 | 0.368 | 0.077 | 0.044 | 0.037 |
|  | Std err. | 0.36136 | 0.137 | 0.511 | 0.508 | 0.507 | 0.216 | 0.176 | 0.183 |  |  |  |
|  | T | 6040 | -4499 | 0.138 | 0.912 | -1298 | -0.824 | 1184 | 2018 |  |  |  |
|  | P | >0.001*** | >0.001*** | 0.891 | 0.369 | 0.204 | 0.417 | 0.246 | 0.053 |  |  |  |
| Percentage vessels occluded | Estimate | 0.023 | 0.130 | 0.044 | 0.243 | -0.023 | -0.105 | 0.124 | -0.051 | 0.030 | 0.009 | 0.012 |
|  | Std err. | 0.204 | 0.078 | 0.288 | 0.286 | 0.286 | 0.122 | 0.099 | 0.103 |  |  |  |
|  | T | 0.112 | 1680 | 0.154 | 0.85 | -0.08 | -0.861 | 1248 | -0.498 |  |  |  |
|  | P | 0.911 | 0.104 | 0.879 | 0.402 | 0.937 | 0.396 | 0.222 | 0.622 |  |  |  |
| Leaf dry mass:Twig dry mass ratio | Estimate | 40569 | -0.918 | -0.599 | -0.136 | -10024 | 0.969 | -0.317 | 0.898 | 0.044 | 0.044 | 0.636 |
|  | Std err. | 0.496 | 0.564 | 0.702 | 0.663 | 0.651 | 0.891 | 0.728 | 0.753 |  |  |  |
|  | T | 8179 | -1628 | -0.853 | -0.205 | -1540 | 1087 | -0.435 | 1192 |  |  |  |
|  | P | >0.001*** | 0.114 | 0.4 | 0.839 | 0.135 | 0.286 | 0.667 | 0.243 |  |  |  |
| Twig dry matter content | Estimate | 4177643 | -113092 | 33787 | -803385 | -245055 | -202133 | 353398 | -0.5601 | 1293 | 1293 | 2251 |
|  | Std err. | 561215 | 335521 | 793678 | 781769 | 778333 | 530505 | 433155 | 448358 |  |  |  |
|  | T | 7444 | -0.337 | 0.043 | -1028 | -0.315 | -0.381 | 0.816 | -0.012 |  |  |  |
|  | P | >0.001*** | 0.738 | 0.966 | 0.313 | 0.755 | 0.706 | 0.421 | 0.99 |  |  |  |
| Leaf area | Estimate | 30257 | -5756 | 11017 | 32462 | 14794 | -7878 | -2259 | 28059 | 399.4 | 399.4 | 275.0 |
|  | Std err. | 29455 | 11727 | 41655 | 41379 | 41300 | 18542 | 15139 | 15670 |  |  |  |
|  | T | 1027 | -0.491 | 0.264 | 0.784 | 0.358 | -0.425 | -0.149 | 1791 |  |  |  |
|  | P | 0.313 | 0.627 | 0.793 | 0.439 | 0.723 | 0.674 | 0.882 | 0.084 |  |  |  |
| Leaf mass per unit area | Estimate | 0.019 | 0.0004 | -0.004 | -0.006 | -0.005 | 0.001 | -0.001 | -0.0003 | 4.071e-06 | 4.071e-06 | 4.071e-06 |
|  | Std err. | 0.003 | 0.001 | 0.004 | 0.004 | 0.004 | 0.002 | 0.002 | 0.002 |  |  |  |
|  | T | 6234 | 0.29 | -1036 | -1525 | -1131 | 0.368 | -0.323 | -0.135 |  |  |  |
|  | P | >0.001*** | 0.774 | 0.309 | 0.138 | 0.267 | 0.716 | 0.749 | 0.894 |  |  |  |
| Leaf dry matter content | Estimate | 644902 | -0.127 | -0.345 | -0.471 | -0.352 | 0.101 | 0.099 | 0.150 | 0.031 | 0.036 | 0.065 |
|  | Std err. | 0.289 | 0.181 | 0.408 | 0.402 | 0.400 | 0.286 | 0.234 | 0.242 |  |  |  |
|  | T | 22331 | -0.7 | -0.845 | -1171 | -0.88 | 0.352 | 0.425 | 0.621 |  |  |  |
|  | P | <2e-16 | 0.49 | 0.405 | 0.251 | 0.386 | 0.727 | 0.674 | 0.539 |  |  |  |
| Leaf thickness | Estimate | 0.372 | -0.071 | -0.096 | -0.096 | -0.050 | 0.053 | 0.041 | 0.075 | >0.001 | >0.001 | >0.001 |
|  | Std err. | 0.028 | 0.013 | 0.039 | 0.039 | 0.038 | 0.021 | 0.017 | 0.017 |  |  |  |
|  | T | 13507 | -5474 | -2462 | -2476 | -1311 | 2597 | 2420 | 4332 |  |  |  |
|  | P | >0.001*** | >0.001*** | 0.020* | 0.019* | 0.200 | 0.015* | 0.022* | >0.001*** |  |  |  |
| Leaf area:Sapwood area ratio | Estimate | -23205 | -0.233 | -0.494 | -0.391 | -0.4647 | 0.4077 | 0.3794 | 0.150 | 0.018 | 0.027 | 0.198 |
|  | Std err. | 0.307 | 0.315 | 0.434 | 0.415 | 0.409 | 0.497 | 0.406 | 0.420 |  |  |  |
|  | T | -7560 | -0.742 | -1138 | -0.943 | -1136 | 0.82 | 0.935 | 0.357 |  |  |  |
|  | P | >0.001*** | 0.464 | 0.264 | 0.353 | 0.265 | 0.419 | 0.358 | 0.724 |  |  |  |
| Pre-dawn leaf water potential | Estimate | 339625 | 220000 | 0.766 | 0.106 | -102054 | -0.675 | -164250 | 0.031 | 0.280 | 0.280 | 0.9864 |
|  | Std err. | 0.899 | 0.702 | 127068 | 123791 | 122839 | 111043 | 0.907 | 0.938 |  |  |  |
|  | T | 3780 | 3133 | 0.603 | 0.086 | -0.831 | -0.607 | -1812 | 0.033 |  |  |  |
|  | P | >0.001*** | 0.004** | 0.552 | 0.932 | 0.413 | 0.548 | 0.080 | 0.974 |  |  |  |
| Mid-day leaf water potential | Estimate | 104050 | 25750 | -12900 | -36104 | -35729 | -22425 | 0.863 | 27466 | 1.367 | 1.367 | 5.408 |
|  | Std err. | 20215 | 16443 | 28589 | 27790 | 27557 | 25999 | 21228 | 21973 |  |  |  |
|  | T | 5147 | 1566 | -0.451 | -1299 | -1297 | -0.863 | 0.406 | 1250 |  |  |  |
|  | P | >0.001*** | 0.128 | 0.655 | 0.204 | 0.205 | 0.395 | 0.687 | 0.221 |  |  |  |

**Table S3.** Principal Component Analysis (PCA) loadings of traits for a PCA ordination (See Figure 5 in main manuscript for PCA biplot).

|  | PCA 1 | PCA 2 | PCA 3 |
| --- | --- | --- | --- |
| Vessel density | 0.097 | 0.116 | -0.160 |
| Theoretical conductivity | 0.238 | 0.374 | 0.593 |
| Vessel grouping | -0.120 | 0.731 | -0.171 |
| Relative vessel fraction | 0.228 | 0.248 | 0.343 |
| Relative parenchya fraction | 0.473 | -0.004 | -0.483 |
| Relative fibre fraction | -0.507 | -0.152 | 0.145 |
| Percentage vessels occluded | -0.452 | 0.343 | -0.010 |
| Leaf thickness | 0.372 | -0.126 | 0.339 |
| Pre-dawn leaf water potentials | -0.193 | -0.189 | 0.141 |
| Mid-day leaf water potentials | -0.089 | -0.241 | 0.294 |
